# State of the Art of Machine Learning–Enabled Clinical Decision Support in Intensive Care Units: Literature Review

**DOI:** 10.2196/28781

**Published:** 2022-03-03

**Authors:** Na Hong, Chun Liu, Jianwei Gao, Lin Han, Fengxiang Chang, Mengchun Gong, Longxiang Su

**Affiliations:** 1 Digital Health China Technologies Ltd Co Beijing China; 2 Department of Critical Care Medicine State Key Laboratory of Complex Severe and Rare Diseases, Peking Union Medical College Hospital Chinese Academy of Medical Science and Peking Union Medical College Beijing China

**Keywords:** machine learning, intensive care units, clinical decision support, prediction model, artificial intelligence, electronic health records

## Abstract

**Background:**

Modern clinical care in intensive care units is full of rich data, and machine learning has great potential to support clinical decision-making. The development of intelligent machine learning–based clinical decision support systems is facing great opportunities and challenges. Clinical decision support systems may directly help clinicians accurately diagnose, predict outcomes, identify risk events, or decide treatments at the point of care.

**Objective:**

We aimed to review the research and application of machine learning–enabled clinical decision support studies in intensive care units to help clinicians, researchers, developers, and policy makers better understand the advantages and limitations of machine learning–supported diagnosis, outcome prediction, risk event identification, and intensive care unit point-of-care recommendations.

**Methods:**

We searched papers published in the PubMed database between January 1980 and October 2020. We defined selection criteria to identify papers that focused on machine learning–enabled clinical decision support studies in intensive care units and reviewed the following aspects: research topics, study cohorts, machine learning models, analysis variables, and evaluation metrics.

**Results:**

A total of 643 papers were collected, and using our selection criteria, 97 studies were found. Studies were categorized into 4 topics—monitoring, detection, and diagnosis (13/97, 13.4%), early identification of clinical events (32/97, 33.0%), outcome prediction and prognosis assessment (46/97, 47.6%), and treatment decision (6/97, 6.2%). Of the 97 papers, 82 (84.5%) studies used data from adult patients, 9 (9.3%) studies used data from pediatric patients, and 6 (6.2%) studies used data from neonates. We found that 65 (67.0%) studies used data from a single center, and 32 (33.0%) studies used a multicenter data set; 88 (90.7%) studies used supervised learning, 3 (3.1%) studies used unsupervised learning, and 6 (6.2%) studies used reinforcement learning. Clinical variable categories, starting with the most frequently used, were demographic (n=74), laboratory values (n=59), vital signs (n=55), scores (n=48), ventilation parameters (n=43), comorbidities (n=27), medications (n=18), outcome (n=14), fluid balance (n=13), nonmedicine therapy (n=10), symptoms (n=7), and medical history (n=4). The most frequently adopted evaluation metrics for clinical data modeling studies included area under the receiver operating characteristic curve (n=61), sensitivity (n=51), specificity (n=41), accuracy (n=29), and positive predictive value (n=23).

**Conclusions:**

Early identification of clinical and outcome prediction and prognosis assessment contributed to approximately 80% of studies included in this review. Using new algorithms to solve intensive care unit clinical problems by developing reinforcement learning, active learning, and time-series analysis methods for clinical decision support will be greater development prospects in the future.

## Introduction

With the popularization of electronic health records, medical equipment, and the improvement of detection methods, patient data are generated in large amounts every day in intensive care units. In traditional clinical data analysis, models and tools can only make use of a limited number of variables in clean and well-organized data. Machine learning has enabled clinical decision support research and applications to generate actionable insights, by utilizing large amounts of intensive care unit patient data, that are useful in many clinical scenarios.

Machine learning, sometimes called the data-driven method, uses statistical analysis models and computational technologies, allowing computer systems to learn from patient data and discover unknown clinical situations. Supervised learning, unsupervised learning, and reinforcement learning are the 3 main types of machine learning [1] used to predict or guide the treatment of patients who are critically ill.

In supervised machine learning tasks, a function maps an input to an output based on example input–output pairs. Functions are inferred from labeled training data. Classification and regression methods, which include but are not limited to linear regression, logistic regression, decision tree, random forest, and support vector machine, are common supervised learning methods.

In unsupervised machine learning tasks, patterns are learned from untagged data. Models are designed to identify or partition large data sets into subsections or clusters that share similar characteristics. In intensive care unit–related tasks, unsupervised learning enables the discovery of latent structures or patient subgroups in specific cohorts [2]. Commonly used unsupervised learning models include clustering, auto-encoding, and principal component analysis.

Reinforcement learning is concerned with how intelligent agents ought to take actions in an environment to maximize the notion of cumulative rewards. The environment is typically defined by a discrete-time stochastic control process called the Markov decision process. In an intensive care unit, clinicians often need to determine treatment plans and make clinical decisions. Reinforcement learning models have great potential for solving these types of problems by providing targeted treatment plans for each patient or patient status and assisting clinicians in making efficient decisions [3-8].

Although there are still challenges when data from multiple sources must be combined, and the performance and ability of machine learning is limited by the volume and quality of data, a number of clinical decision support studies [9,10] have demonstrated the ability to use sophisticated machine learning models to solve certain intensive care unit–related tasks, and their performance has been shown to be comparable with human abilities, and for certain tasks, even it potentially exceeds human abilities [7,11].

We sought to focus on machine learning research and applications adapted to clinical decision support in intensive care units, which may directly help clinicians diagnoses accurately, predict outcomes, identify risk events, or decide treatments at the intensive care unit point of care.

## Methods

### Search Strategy

We searched for papers in the PubMed database that had been published prior to October 2020 using a query combination of MeSH terms (“intensive care unit,” “critical care,” “machine learning,” “artificial intelligence,” “decision support systems, clinical”) and keywords in the title or abstract keywords related to *machine learning* (“machine learning,” “artificial intelligence,” “prediction model,” “predictive model,” “predictive modeling,” “artificial learning,” “predictive analysis,” “machine intelligence,” “data driven,” “data-driven,” “statistical learning,” “neural network,” “deep learning,” “reinforcement learning,” “time series,” “time-series,” “algorithm”), *decision-making* (“clinical decision support system,” “medical decision,” “decision tool,” “support tool,” “clinical decision,” “physician decision,” “clinician decision,” “decision algorithm,” “CDSS,” “CDS,” “clinical management,” “decision making,” “decision-making“), and *intensive care units* (“intensive care,” “ICU,” “critical care,” “intensive care unit”).

### Selection Criteria

We included English-language papers that reported studies (both prospective and retrospective studies) on clinical decision support, with machine learning methods that targeted a specific clinical scenario of intensive care units. We excluded papers that were systematic reviews and meta-analyses, studies of clinical decision support system implementations or clinical decision support system usability evaluations, studies that described rule-based clinical decision support system, studies that used data that were not from patients in intensive care units (eg, studies for intensive care unit admission prediction but using patient data from other departments, such as emergency or surgery departments), studies with outcomes irrelevant to regular intensive care unit clinical care (eg, studies about estimation of caffeine regimens), and studies that did not use machine learning methods (eg, studies using clinical scores or statistical analysis on small samples).

### Data Analysis

We extracted the following information from selected papers for content analysis: study cohort, machine learning models, analysis variables, evaluation methods, and research topics.

#### Study Cohort

In general, the greater the number of data sets to which a machine learning model is applied, the stronger its generalization capabilities. Therefore, we investigated the inclusion cohorts and distribution centers of each study and classified these studies into single-site or multisite studies accordingly. We also classified studies by *c*, the sample size of studies: *c*<500, 500<*c*<2000, 2000<*c*<5000, 5000<*c*<10,000, 10,000<*c*<50,000, and *c*>50,000.

#### Machine Learning Models

The model methods or algorithms used in each paper were reviewed for analysis, and model methods were categorized as supervised learning, unsupervised learning, or reinforcement learning.

We reviewed variables or features used for modeling in each study. According to routine intensive care unit practices, we classified these variables into 12 groups: demographic variables, vital signs, symptoms, laboratory values, ventilation parameters, medications, nonmedicine therapy, comorbidities, fluid balance, scores, medical history, and outcome. Given the wide range of variable expressions in papers, such as formal medical terms, abbreviations, acronyms, and capitalizations, variable name normalization was implemented using text processing and manual annotation methods. As some studies used self-defined features or derived data for their special study purpose, variables used in only 1 study were excluded.

#### Evaluation Methods

To determine the applicability and potential impact of various machine learning models for clinicians and patients (ie, in applications), model evaluation methods are important components of model development. We reviewed evaluation metrics used for measuring model performance.

#### Research Topics

In addition to overall quantitative analysis, which included all studies, selected papers were divided into 4 topics for detailed analysis: detection and monitoring for diagnosis, early identification of clinical events, patient outcome prediction, and treatment decisions.

## Results

### General

**Figure 1:**
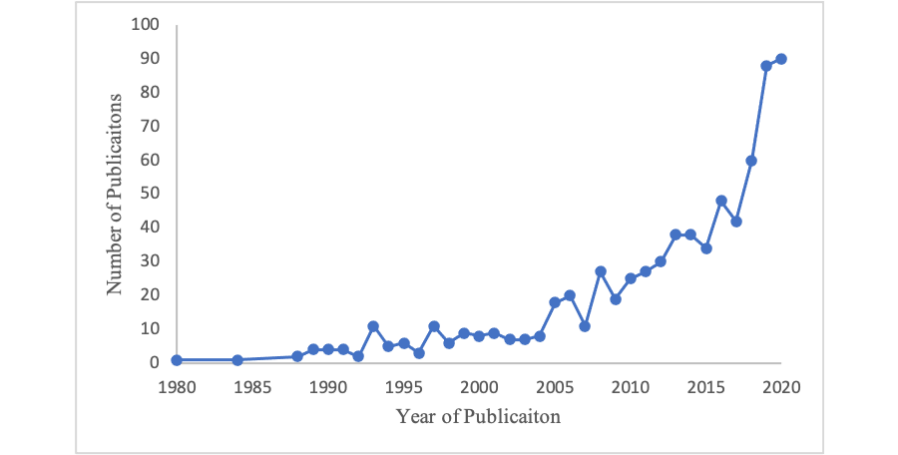
Growth in number of publications.

A total of 643 papers were found. The number of machine learning–enabled intensive care unit clinical decision support system research papers published in the PubMed database has been continuously increasing between January 1980 and October 2020 (Figure 1).

**Figure 2:**
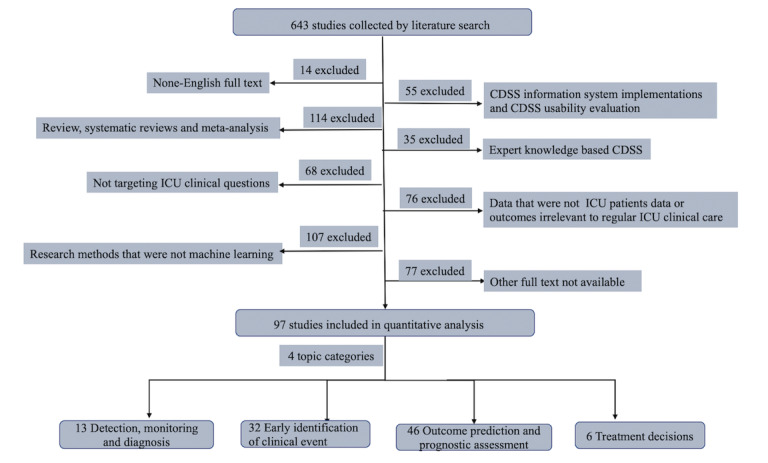
Article review process. CDSS: clinical decision support system; ICU: intensive care unit.

Among the 643 papers identified and assessed for eligibility, 14 non–English language papers, 55 clinical decision support system implementations and clinical decision support system usability evaluations, 114 reviews and meta-analyses, 35 expert system clinical decision support system studies, 68 studies not about intensive care unit clinical questions, 76 studies using patient data from other clinical departments or with outcomes irrelevant to regular intensive care unit clinical care, 107 studies that used methods other than machine learning, and 77 studies for which full-text papers were unavailable were excluded (Figure 2); therefore, 97 papers remained (Table 1).

Most studies used data from adult patients (n=82, 84.5%); however, 8 studies used data from pediatric patients (8.2%) and 7 studies used data from neonates (7.2%). Two-thirds of the studies (65/97, 67.0%) were developed from single-center data sets, and 32 (33.0%) were developed from a multicenter data set; cohort sizes also varied (*c*<500: 35/97, 36%; 500<*c*<2000: 19/97, 20%; 2000<*c*<5000: 12/97, 12%; 5000<*c*<10000: 10/97, 10%; 10000<*c*<50000: 16/97, 16%; *c*>50,000: 7/97, 7%).

**Figure 3:**
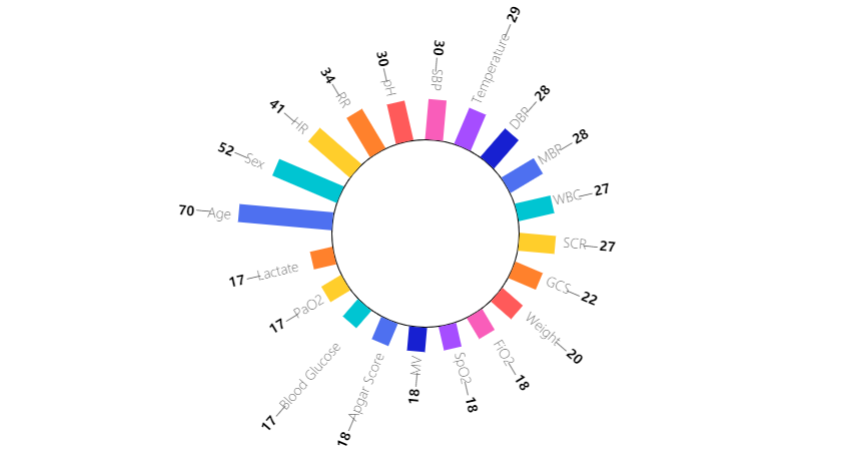
Top 20 most frequently used variables. DBP: diastolic blood pressure; FiO2: fractional inspired oxygen; GCS: Glasgow Coma Scale; HR: heart rate; MBP: mean blood pressure; MV: mechanical ventilation; PaO2-partial pressure of oxygen; RR: respiratory rate; SBP: systolic blood pressure; SCR: creatine; SpO2: peripheral capillary oxygen saturation; WBC: white blood cell count.

The vast majority of studies used supervised learning (88/97, 91%), and only a few used unsupervised learning (3/97, 3%) or reinforcement learning (6/97, 6%). In total, 849 variables for model analysis were extracted. The most frequent variable categories are shown in Table 1, and the top 20 most frequently used variables are shown in Figure 3.

Most studies used more than 1 evaluation metric. The most frequently used were area under receiver operating characteristic curve (n=57), sensitivity (n=37), specificity (n=31), and accuracy (n=24).

**Table 1:** General characteristics of the selected studies.

Characteristic			Value (n=97), n
**Types of decision support**			
	Detection, monitoring, and diagnosis	13	
	Early identification of clinical events	32	
	Outcome prediction and prognostic assessment	46	
	Treatment decisions	6	
**Population**			
	Adult	82	
	Pediatric patients	8	
	Neonates	7	
**Medical setting**			
	Single-center	65	
	Multicenter	32	
**Type of machine learning**			
	Supervised learning	88	
	Unsupervised learning	3	
	Reinforcement learning	6	
**Type of variables**			
	Demographic variables	74	
	Laboratory values	59	
	Vital signs	55	
	Scores	48	
	Ventilation parameters	43	
	Comorbidities	27	
	Medications	18	
	Outcome	14	
	Fluid balance	13	
	Nonmedicine therapy	10	
	Symptoms	7	
	Medical history	4	
**Type of evaluation method, n^a^**			
	Area under the receiver operating characteristic curve	57	
	Sensitivity	37	
	Specificity	31	
	Accuracy	24	
	Positive predictive value	11	

^a^More than 1 variable type could be used in each study.

### Monitoring, Detection, and Diagnosis

#### Overview

Among 13 studies, 4 (30.8%) studies [12-15] focused on monitoring or detection of physiological indicators, 3 studies (23.1%) [16-18] focused on the of mechanical ventilation abnormalities (in particular, patient-ventilator asynchrony), 4 studies (30.8%) [19-22] used electroencephalography (EEG) to diagnose brain diseases, and 2 studies (15.4%) [11,23] studies focused on infections. Variables used included demographic variables (n=5), vital signs (n=6), laboratory values (n=5), ventilation parameters (n=5), comorbidities (n=1), and outcome (n=1).

Most data were obtained from a single center (11/13, 84.6%), and only 2 studies (2/13, 15.4%) used multicenter data sets. Some studies (3/13, 23.1%) used data from public databases, such as the MIMIC database, the public NIH Chest-XRay14, and PLCO data sets (Multimedia Appendix 1).

The top 3 models used were neural network (n=4), tree (n=3), and random forest (n=3) models. Support vector machine models were used twice (n=2). Other models, such as logistic regression, and linear regression were only used in 1 study each.

Model performance was mainly evaluated with sensitivity (n=7), specificity (n=8), area under the receiver operating characteristic curve (n=3), and accuracy (n=3), whereas other evaluation methods such as equal error rates, F1 score, recall, and κ coefficients were each used only once.

#### Monitoring of Physiological Indicators

Quinn et al [13] provided a general model for inferring hidden factors from clinical data and was successfully applied to the major task of monitoring premature infants in the intensive care unit. Eshelman et al [12] described an algorithm consisting of a set of rules for identifying intensive care unit patients who may become hemodynamically unstable. Taking into account the individual differences of intensive care unit patients, Zhang and Szolovits [15] developed an algorithm based on personalized vital signs data to improve the accuracy of alarms. Charbonnier [14] extracted online temporal episodes from the high-frequency physiological parameters of intensive care unit patients to visually support signal interpretation.

#### Mechanical Ventilation

Mechanical ventilation is widely used in intensive care units, during which a series of parameters need to be monitored. Kwok et al [16] established a nonlinear adaptive neuro-fuzzy inference system model for fractional inspired oxygen estimation, which reduced the need for invasive inspections. Two groups of researchers discussed the problem of patient-ventilator asynchrony, and developed a classifier based on machine learning to detect abnormal waveforms [17,18].

#### Electroencephalography Monitoring

EEG monitoring plays an important role in the detection of brain function and the diagnosis of brain disease. Koolen et al [19] developed a method for the automated classification of neonatal sleep states via EEG. Golmohammadi et al [21] presented a system that can achieve high-performance classification of EEG events that might correlate with epilepsy, metabolic encephalopathy, cerebral hypoxia, and ischemia. Farzaneh et al [20] developed a machine learning framework to automatically segment and assess the severity of patients with subdural hematoma during traumatic brain injuries [20].

#### Diagnosis of Infection

Infections are an important clinical issue in intensive care. Sepsis is a common and serious condition in the intensive care unit that results from an overreaction to infection that damages tissues and organs and can lead to complications, making it one of the leading causes of hospital-related deaths [24]. A high-performance algorithm, InSight, was demonstrated to be superior to the commonly used Modified Early Warning Score, Simplified Acute Physiology Score, and Systemic Inflammatory Response Syndrome score for the diagnosis of patients with alcohol use disorder combined with sepsis shock [23]. In addition, it is still challenging to explain lung opacity in radiography of the supine chest of patients with lung infection in the intensive care unit—Rueckel et al [11] evaluated a prototype artificial intelligence algorithm that could classify underlying lung opacity, which might suggest a diagnosis pneumonia.

### Early Identification or Prediction of Clinical Events

#### Overview

Clinical event prediction, the use of data from electronic health records to predict the occurrence of certain events or the best time to give treatment, is one of the most important aspects of intensive care unit clinical decision support system. Among 32 clinical event prediction studies, 3 (9.4%) were related to acute kidney injury, 11 (34.4%) were related to infection prediction, 8 (25%) were related to respiratory diseases, and 10 (31.3%) were related to other predictions and evaluations (Multimedia Appendix 1).

In intensive care unit clinical prediction and evaluation studies, up to 87 variables were used in a single paper. Categories of variables, in order of frequency, were laboratory values (n=25), demographic variables (n=25), vital signs (n=20), scores (n=18), ventilation parameters (n=14), fluid balance (n=8), medications (n=7), comorbidities (n=7), outcome (n=4), nonmedicine therapy (n=3), symptoms (n=3), and medical history (n=1).

More than three-quarters of the studies (25/32, 78%) were based on data from a single center, 10 of which were from the freely available public database Medical Information Mart for Intensive Care II or III. Multi-institutional data were used in the other studies (7/32, 22%).

Logistic regression was the most commonly used method (11/32, 34%), followed by neural networks (7/32, 21%), and random forest (6, 19%). Support vector machine and decision tree models were each used in 5 (15.6%) studies. Naive Bayes, gradient boosting tree model, extreme gradient boosting, fuzzy model, and Insight each appeared twice (6.3%).

Sensitivity (n=16) and area under receiver operating characteristic curve (n=17) were the most commonly used evaluation metrics, followed by specificity (n=12) and accuracy (n=12). The following metrics appeared in fewer than 10 papers: positive predictive value (n=3), F1 score (n=4), and mean absolute error (n=2).

#### Acute Kidney Injury Prediction

Early prediction of acute kidney injury has a high value for the long-term survival and quality of life of critically ill patients. Acute kidney injury is often associated with high morbidity and mortality rates in intensive care units. The status of other vital organs, initiation of therapy, patient response, and preexisting comorbidities can all contribute to the development of acute kidney injury [25]. Multiple machine learning methods have been utilized and compared to analyze unstructured clinical records and structured physiological measurements to identify early episodes of acute kidney injury [26]. Soliman et al [25] studied the prognostic impact of early acute kidney injury predicted by data from the first day of admission. One study [27] focused on patients younger than 21 years, who are more likely to recover from disease.

#### Prediction of Sepsis and Infection

Early identification and treatment is the key to survival for many sepsis and infection patients [28], but it is difficult for clinicians to predict before it occurs, because it is extremely complex and each patient is different. Early prediction of sepsis using interpretable or uninterpretable machine learning models can help clinicians enhance the accuracy of fever workup [28] to identify and intervene in a timely manner [29-33]. One research aim is to make accurate predictions with as little electronic health record data as possible [34]. Mao et al achieved early prediction of sepsis using only vital signs validated in multiple centers [35]. The prediction of neonatal sepsis has also received substantial research attention in recent years [36,37]. One paper [38] focuses on predicting infections caused by a specific microorganism—invasive fungal disease due to *Candida* species—in intensive care unit patients.

#### Prediction of Respiratory Disease and Mechanical Ventilation

Respiratory management in the intensive care unit is an important aspect of critical care and treatment. Early diagnosis of respiratory critical illness has a significant impact on patient prognosis [39]. In addition, maintenance of cardiopulmonary function is required in patients admitted to the intensive care unit due to acute symptoms such as direct trauma, pulmonary infection, heart failure, and sepsis. Machine learning methods can help predict the onset of acute respiratory disease in patients, especially in pediatric patients. Sauthier et al [40] used random forest and logistic regression to predict the time of acute hypoxic respiratory failure in critically ill children with severe influenza. Messinger et al [39] applied a cascaded artificial neural network to design new respiratory scores for early identification of asthma in young children. In addition, early prediction of acute respiratory distress syndrome was studied because of its high morbidity and mortality [41].

Furthermore, ventilator weaning and reintubation after weaning are currently well studied [42,43] in intensive care unit clinical decision support system literature, as well as the effect of drugs on intubation [44]. Moreover, predicting patient oxygen saturation after ventilation [45] and risk factors for failure of mechanical ventilation [46] can help health care professionals respond in a time manner.

#### Other Predictions and Evaluations

There were 10 papers that could not be classified; we simply put them into one class separately. There were forecasts for detection and monitoring indicators, such as urine output after fluid administration [47], glucose [48], lactic acid [49], and activated partial thromboplastin time [50]. Lin [47] established a gradient tree-based machine learning model implemented with extreme gradient boosting algorithms to predict urine output in sepsis patients after fluid resuscitation to prevent fluid overload-related complications. Pappada et al [48,49] developed a neural network–based model to obtain a complete trajectory of glucose values up to 135 minutes in advance. Mamandipoor et al [49] combined least absolute shrinkage and selection operator regression, random forest, and long short-term memory to predict blood lactate concentration in patients in the intensive care unit. Our previous study also compared multiple machine learning approaches to guide clinical heparin administration by predicting the range of activated partial thromboplastin time values [50]. There were also studies that aimed to reduce unnecessary laboratory tests to streamline the process and reduce the burden on patients [51,52]. Predicted clinical events also included acute traumatic coagulopathy [53], delirium [54], advanced anemia [55], and fluid resuscitation therapy [56].

### Outcome Evaluation and Prognostic Assessment

#### Overview

Of 46 papers that used machine learning for outcome evaluation for patients who were critically ill, 11 papers (23.9%) predicted overall mortality and survival, 23 papers (50%) predicted the outcomes of patients with certain diseases, and 12 papers (26.1%) included treatment prognosis, length of stay in the intensive care unit, and other outcome evaluations (Multimedia Appendix 1).

Categories of variables, in order of frequency, were demographic variables (n=39), scores (n=24), laboratory values (n=23), ventilation parameters (n=20), vital signs (n=18), comorbidities (n=17), medications (n=10), outcome (n=8), nonmedicine therapy (n=7), fluid balance (n=4), symptoms (n=4), and medical history (n=3).

Of the 46 outcome prediction studies, 25 (54.3%) were based on single-center data, 6 of which used data from MIMIC II and III, and the other 21 studies (45.7%) made use of multicenter data.

Logistic regression was the most commonly used method (27/46, 59%), followed by random forest (9/46, 20%), random forest (8/46, 17%), support vector machine (7/46, 15.2%) and decision tree model (5/46, 11%) studies. The gradient boosting tree model appeared in 4 (9%) studies, and adaptive boosting and linear regression each appeared twice (4.3%). Other models that appeared only once are not discussed here.

Area under receiver operating characteristic curve (n=37) was the evaluation metric used most often, followed by sensitivity (n=14), specificity (n=11), positive predictive value (n=4), accuracy (n=8), negative predictive value (n=6),F1 score (n=2), Matthews correlation coefficient (n=2), and Brier score (n=2).

#### Overall Intensive Care Unit Patient Outcomes

Typical outcomes were overall mortality [57-62], survival [63], and long-term quality of life [64]. Mortality [65,66] and survival status at 1 year [67] in critically ill patients aged 80 years and older were also studied using machine learning methods.

#### Outcomes of Patients With Specific Diseases

Patients with sepsis and infection remain one of the most studied populations in terms of mortality (generally 28 days) [68-72], followed by acute kidney injury [72-75]. There is an increasing trend in outcome prediction studies in critically ill patients with liver disease—acute liver injury [76,77], cirrhosis [77], and advanced liver disease [78] have been studied using machine learning. In patients with severe cancer, 30- [79] and 120-day [80] survival rates were studied retrospectively with logistic regression models.

For cardiac disease, Lee et al [81] used EEG data to predict the outcome of children with cardiac arrest and Murtuza et al [82] found that arterial blood lactate levels can be associated with mortality in children who have undergone cardiac surgery. For brain diseases, the outcomes of patients with subarachnoid hemorrhage [83] and severe traumatic brain injury [84] have been analyzed. Wildman et al [85] predicted the impact of chronic obstructive pulmonary disease and asthma on mortality in critically ill patients. Daly et al [86] used logistic regression to study the relationship between early discharge and mortality with the intention of reducing mortality in this group of intensive care unit patients. Other papers [87-89] examined patient outcomes and factors influencing them after deterioration. Ebadollahi et al [90] predicted the temporal trajectory of physiological data with patient similarity, with the aim to identify universal patterns of disease progression from a large amount of clinical practice data, to establish a generalized computer-aided clinical decision support framework for personalized treatment.

#### Treatment Prognosis and Intensive Care Unit Stay Time Evaluation

Evaluating the outcome of certain treatments through machine learning can help medical professionals refine their treatments to achieve better therapeutic effects. Evaluation of outcomes after extubation based on continuous vital sign information and static characteristics of children can help adjust the timing of extubation to reduce mortality [91-93]. Evaluation of prolonged mechanical ventilation [94] and 1-year and 5-year functional survival [95] after cardiac surgery was used to help adjust and optimize postsurgical care practices. Evaluating the length of stay in the intensive care unit [96,97] and the risk of readmission after discharge from the intensive care unit [98] to effectively forecast the trend of the disease could improve treatment and care. In addition, designing and improving critical illness scores to indicate disease severity [99-101] was studied. For example, McRae et al [102] designed a score to quickly determine the severity of COVID-19 and achieved optimistic results in 160 individuals.

### Treatment Decisions

Treatments, clinical determination, and decision-making in the intensive care unit were studied in 6 papers [3-8]. These papers focused on various clinical questions and mainly used a reinforcement learning model. Among them, 4 papers [3,5,7,8] (67%) addressed drug dosage, such as optimal vasopressin dose [3,7], heparin dosage [5], and morphine dosage [8]. The other 2 papers [4,6] (33%) studied the timing of mechanical ventilation extubation.

Categories of variables, in order of frequency, were vital signs (n=6), demographic variables (n=5), laboratory values (n=5), ventilation parameters (n=3), medications (n=4), fluid balance (n=2), scores (n=4), and comorbidities (n=1) (Multimedia Appendix 1).

Reinforcement learning models can be divided into conventional reinforcement learning models (that is, wherein the reward function is known and we only need to find a policy to maximize the reward function) and inverse reinforcement learning models (that is, wherein the reward function is unknown, and we have to learn the most reasonable reward function through the decision-making examples of clinicians)—4 papers used typical reinforcement learning model, and 2 papers used inverse reinforcement learning models.

All 6 papers used patient data from the intensive care units in US hospitals. Most papers used single-center data from MIMIC II (n=1) or MIMIC III (n=4), with *c* ranging from 707 to 96,156 (mean 22,256; median 7852).

Because the output of a reinforcement learning model is a policy that is not easy to evaluate, in these studies, the policy given by the model was compared with that actually given by the doctor; when the 2 policies differed, the effect of the reinforcement learning model was analyzed according to the actual clinical problem.

## Discussion

From reviewed studies, we concluded that early identification of clinical outcome prediction and prognosis assessment contributed to approximately 80% of studies, and machine learning–based clinical decision support applications in intensive care unit could support timely bedside decision-making [15], transform data into more actionable insights or evidence-based clinical rules [101], assist disease diagnosis [30], predict adverse outcomes before they happen [76], enable continuous assessment of patient responses to critical care interventions [91], allow better management of highly complex situations and the best treatment decisions [3], ultimately reduce clinicians burden [52], and allow clinicians to have more time to deliver their knowledge, experience, and human care in practice [64].

We found that 91% (88/97) of reviewed studies used supervised learning methods. Unsupervised learning is commonly used for phenotyping or patient subgrouping [2], usually to discover new knowledge; therefore, explaining and validating subgroups or patterns with reasonable clinical meaning is a challenge. Reinforcement learning models have great potential for solving medical decision problems; however, to the best of our knowledge, there is a lack of sophisticated reinforcement learning models to guide intensive care unit decision-making [5]. Data-driven decision support tools will permit clinicians to function more efficiently, caring for more patients more safely; however the selection of a model should be tailored to the clinical scenario [9,10]; therefore, we need a better understanding of which algorithms are a best fit for which clinical scenarios.

We also found that many machine learning–based clinical prediction tasks are still challenging. First, not all the data collected from intensive care unit are good quality data or complete [7], particularly when data from different sources were included in one predictive model. Various data in the intensive care unit include general available data in the electronic health record, such as patient information, encounter information, diagnoses, intervention, routine laboratory data, imaging, natural language and physiologic data, as well as limited available information in the intensive care unit, such as social information, omics data, pathology, radiology, and wearable data [103]. This makes data preprocessing a difficult and time-consuming task. Second, parameter optimization was used to obtain the best parameter combination to improve model accuracy. Model parameters need to be determined and fitted using the training data set, and many adjustable hyperparameters must be tuned to obtain a model with optimal performance [104]. Generally, the more complex the model, the more parameters need to be adjusted, and the more difficult it is to adjust the parameters. For example, in logistic regression [74], usually only the regularization coefficient is adjusted; and in random forest models [53], the hyperparameters that need to be adjusted include the number of trees, the maximum depth of the tree, and the split criteria. Third, typically, the more complex the model, the higher the required sample size [105]. If the sample size is insufficient, overfitting occurs easily, which leads to instability or inaccuracy of the model. In some clinical scenarios, owing to the limited sample size, the use of complex models is limited [59]. Last, after developing the model, prospective evaluation using external data sets and clinical trials should be conducted before using the model in practice [106] to improve confidence in machine learning predictions [7]; however, performing strong validation of a machine learning model’s generalizability and interpretability is challenging; internal validation approaches, such as cross-validation and bootstrapping, cannot guarantee the quality of a machine learning model due to potentially biased training data and the complexity of the validation procedure itself [107]. Lack of technical and semantic interoperability makes harmonization of patient data from one center to another costly. As inconsistent model results may be derived when adapting to new data sets [108], retraining models using data from other sources would minimize the cost and allow models to incorporate new clinical settings.

Future research should expand the innovation and exploration using new algorithms to solve intensive care unit clinical problems by developing reinforcement learning, active learning, and time-series analysis methods for clinical decision support. In addition, machine learning modeling requires recognition, understanding, and trust from intensive care unit clinicians. Model developers must provide full explanations of modeling methods, input, output, experimental and trial settings, clinical scenarios, and operation methods to clinicians. With the basis to understand, operate, and debug the outputs of a model, clinicians can have more confidence in accepting the model results and take action on the basis of that model’s recommendations.

## Appendix

Multimedia Appendix 1 Supplementary information.

## Abbreviations

EEG: electroencephalography
