# Supplementary material for: State of the Art of Machine Learning–Enabled Clinical Decision Support in Intensive Care Units: Literature Review
Source: JMIR Med Inform. 2022 Mar 3;10(3):e28781. doi: 10.2196/28781 (PMC8931648; doi:10.2196/28781)
Supplement: Multimedia Appendix 1 [file medinform_v10i3e28781_app1.docx]

### Appendix I: Monitoring, Detection, and Diagnosis

| **Article Refs** | **Learning Models** | **Variables** | **Cohort Size** | **Single center/ Multi center** | **Infants/Children/Adults** | **Evaluation Methods** |
| --- | --- | --- | --- | --- | --- | --- |
| Eshelman LJ, et al.[12] | support vector machines (SVM) and neural nets (NN) | BUN(blood urea nitrogen), WBC (white blood cellcount), PTT (partial thromboplastin time), hematocrit, HR, systolic BP (arterial if available, otherwise noninvasive), and OxI (oxygenation index = Fraction of Inspired Oxygen*Mean Airway Presssure/PaO2). | 12695 | Single center, MIMIC II | Adults | sensitivity and specificity |
| Quinn JA, et al.[13] | (F)SLDS, GS, RBPF, FHMM | Core body temperature(°C), Diastolic blood pressure(mmHg), Heart rate(bpm), Peripheral body temperature(C), Saturation of oxygen in pulse(%), Systolic blood pressure(mmHg), Transcutaneous partial pressure of CO2(kPa), Transcutaneous partial pressure of O2(kPa), | 15 | Single center | Infants | AUC, EER |
| Charbonnier S, et al.[14] | decision tree | hemodynamic parameters  (systolic, mean and diastolic blood pressures, heart  rate, oxygen saturation) or respiratory parameters  (volume expired, respiratory rate, minute ventilation, maximal pressure in the airways). | 10 | Multi center | Adults | External observer evaluation |
| Zhang Y, et al.[15] | Classification tree and neural network learning | heart rate derived from ECG waveforms, pulse rate fromplethysmography, respiration rate, blood pressure (systolic, diastolic and mean), either arterial or measured by non-invasive means, arterial and venous oxygen saturation, and oxygen perfusion | 11 | Single center | Children | sensitivity, specificity, positive predictive value, and accuracy |
| Kwok HF, et al.[16] | A linear regression model and a non-linear adaptive neuro-fuzzy inference system (ANFIS) model | age, gender, weight, height, body temperature, Hb, arterial pH, bicarbonate concentration, CO and VO2 , FiO2, positive end-expiratory pressure (PEEP), ventilatory rate (RR), tidal volume, peak inspiratory pressure (PIP), inspiratory time and inspiratory to expiratory ratio (I:E ratio) | 40（202data sets） | Single center | Adults | Themean squared controlerror |
| Rehm GB, et al.[17] | (i) Random Forest (RF), (ii) Multilayer Perceptron (MLP), (iii) Extremely Randomized Trees classifier (ERTC), (iv) Gradient Boosted classifier (GBC) | TVi, TVe, TVe/TVi, I-time, E-time, I:E ratio, RR | 35patients, 9719 breaths | Single center | Adults | true positive,  true negative, false positive, and false negative counts, sensitivity and specificity |
| Gholami B, et al.[18] | Random Forests | mechanical ventilation waveform | 11 | Single center | Adults | sensitivity, specificity, the kappa coefficients |
| Koolen N, et al.[19] | support vector machine classifier | Patients (N) , Gender (M/F) , PSGs per patient , Number of all PSG studies , Birth Weight (g) , Gestational Age (wks) , Postmenstrual Age (wks) , IVH any 14, IVH severe (III-III+) | 231 EEG recordings from 67 infants | Single center | Infants | accuracy, sensitivity, specificity |
| Farzaneh N, et al.[20] | deep convolutional neural network model, random forest model | Age, Radial distance, Azimuth angle, Elevation angle, Distance to skull, Minimum, Maximum, Average, Standard deviation, Skewness, Kurtosis, Entropy, Gabor, Laplacian of Gaussian | 110 | Single center | Adults | F1, recall, specificity |
| Golmohammadi M, et al.[21] | hidden Markov models (HMMs), deep learning | EEG recordings | 518 | Single center | Adults | Sensitivity, Specificity,  DET curve |
| Sorani MD, et al.[22] | hierarchical clustering | MAP, ABP-systolic, ABP-diastolic, ICP ETCO, SvO, HR, CPP, SpO2, Core Temp PaO2, Brain Temp, Plateau pressure, PEEP breathing pressure, Peak breathing pressure, Tidal volume, Spontaneous minute volume, Minute ventilation, Respiratory rate , Inspired O2 | 23 | Single center | Adults | Heat map |
| Rueckel J, et al.[11] | deep neural network | Age, Sex, C-reactive protein, Intubated at the time of image acquisition, Tracheal tube inserted, Pneumonia in clinical supine chest radiograph, underlying primary disease, etc. | 166 | Multi center，the public NIH Chest-XRay14 and PLCO dataset | Adults | receiver-operating characteristic curve, sensitivity, specificity, positive predictive value, negative predictive value, and accuracy |
| Calvert J, et al.[23] | logistic regression | blood oxygen saturation, heart rate, pH, pulse pressure, respiration rate, systolic blood pressure, temperature, and white blood cell count. | 29, 083 | Single center, MIMIC III | Adults | AUROC |

### Appendix II: Early Identification or Prediction of Clinical Events

| **Article Refs** | **Learning Models** | **Variables** | **Cohort Size** | **Single center/ Multi center** | **Infants/Children/Adults** | **Evaluation Methods** |
| --- | --- | --- | --- | --- | --- | --- |
| Sun M, et al.[26] | Logistic regression (LR), random forest (RF), multinomial naïve Bayes (NB), supported vector machine (SVM) classifiers and mixed-feature Convolutional Neural Network | Age, Gender, Ethnicity, Heart rate maximum, Heart rate mean, Systolic BP minimum, Systolic BP mean, Diastolic BP minimum, Diastolic BP mean, Temperature maximum, SpO2 minimum, SpO2 mean, Glucose level maximum, Bicarbonate level minimum, Creatinine level minimum, Creatinine level maximum, Hemoglobin level minimum, Platelet count minimum, Potassium level maximum, Partial thromboplastin time minimum, Partial thromboplastin time maximum, International normalized ratio minimum, International normalized ratio maximum, Prothrombin time minimum, Prothrombin time maximum, Blood urea nitrogen level maximum, White blood cell count maximum, Calcium level minimum, Average urine output, Estimated glomerular filtration rate, Mechanical Ventilation | 14, 469 | Single center, MIMIC III | Adults | AUC |
| Soliman IW, et al.[25] | multivariable Poisson regression model | serum creatinine, urine output per hour within the first 24 hours, age, sex, pre-ICU hospital length of stay, admission type,  Charlson Comorbidity Index, the need for mechanical ventilation, confirmed infection, the acute physiology score (APS, as part of the APACHE II score) | 2420 | Single center | Adults | RR 95% CI, P value |
| Sanchez-Pinto LN, et al.[27] | multivariate linear regression models | UIb, VASb, age, BMI, Charlson co-morbidity index,  hospital days prior ICU admission, APACHE II, SOFA, TISS-28, NEMS, sub oncological, sub hematological, sub cirrhosis, sub elderly (≥80 years),  gender, previous hospitalization in the past 6 months, admission during weekend, admission unplanned, DNR, MV, VP, RRT, surgery, medical imaging, tracheotomy, transfusion, living situation at baseline, ADL, origin of hospital admission, origin of ICU admission, baseline work, main ICU diagnosis | 1, 953 | Single center | Adults | R2(proportion of explained variance), adjust R2, the root of the cross-validated prediction error |
| Fadlalla AM, et al.[28] | decision trees, neural networks, and logistic regression analysis models | Intensive care unit stay, Age, Max. body temperature, Max. leukocyte count, Injury Severity Score, Sex, Mechanism of injury, Presence of a central line, Presence of mechanical ventilation, Use of antibiotics the day prior to cultures, Fever or leukocytosis | 510 | Single center | Adults | sensitivity, accuracy, discrimination |
| Nemati S, et al.[29] | a modified Weibull-Cox proportional hazards model | age, gender, ethnicity, Mean Arterial Pressure (MAP), Heart Rate (HR), Peripheral capillary Oxygen Saturation (SpO2), Systolic Blood Pressure (SBP), Diastolic Blood Pressure (DBP), Respiation Rate (RESP), etc. | 27, 000 / 42, 000 | Multi center | Adults | AUROC, prediction task (tsepsis, tSOFA, tonset) and prediction window (n = 4, 6, 8, and 12 hr), sensitivity, Accuracy |
| Kam HJ, et al.[30] | InSight, Deep feedforward networks, LSTM | systolic blood pressure, pulse pressure, heart rate, body temperature, respiration rate, white blood cell count, pH, blood oxygen saturation, age | 5, 789 | Single center, MIMIC II | Adults | AUROC, Sensitivity, Specificity, Accuracy |
| Kaji DA, et al.[31] | LSTM | RBCs, WBCs, platelets, hemoglobin, hemocrit, atypical lymphocytes, bands, basophils, etc. | 56841 | Single center, MIMIC-III | Adults | auroc, positive predictive value, sensitivity |
| Scherpf M, , et al.[32] | recurrent neural network | patient age, systolic blood pressure, diastolic blood pressure, pH value, blood oxygen saturation (SO2), temperature, heart rate, respiratory rate, CO2 partial pressure(PaCO2), white blood cell count, etc. | 34334 | Single center, MIMIC III | Adults | AUROC, sensitivity |
| Wang SL, et al.[33] | SVM | age, gender, cause of sepsis, IL-6, CRP, historical outcomes (mild or severe) | 1000 | Single center | Adults | sensitivity, specificity, accuracy, auroc |
| Desautels T, et al.[34] | InSight | ICU type, Gender, Age, Length of stay , Death during hospital stay, GCS, Heart rate, Respiration rate, Spo2, Temperature, NIDiasABP, NISysABP, SysABP, DiasABP | 22, 853 | Single center, MIMIC III | Adults | area under the receiver operating characteristic curves (AUROC) and area under precision-recall curves (APR), F1, DOR |
| Mao Q, et al.[35] | gradient tree boosting | Gender, Age, Length of stay (days) in ICU, Death during hospital stay, ICD-9 code, Gold standard, InSight (95%CI), InSight label defnitions removed (95%CI), MEWS, SOFA, SIRS | 111, 957 | Multi center | Adults | AUROC, Sensitivity, Specificity |
| Metsvaht T, et al.[36] | stepwise multiple logistic regression (MLR) analysis, classification, regression tree analysis | blood cell count (WBC), differential and ratio of immature to total neutrophil count (I/T ratio), C-reactive protein (CRP), serum glucose and total bilirubin, serum albumin, creatinine, urea and liver function tests(LFT) , birth weight (BW) , gestational age (GA), first and fifth minute Apgar score, need for respiratory support in the delivery room, age at intubation, surfactant administration, need and duration of sustained respiratory support, age on admission to NICU, time and type of initial and subsequent AB regimens, need for vasoactive therapy within the first three days of life with number of agents used, intolerance of enteral feeding, defined as less than 10% of total calories supplied via the enteral route | 283 | Single center | Infants | Sensitivity, Specificity, Positive predictive value, Negative predictive value |
| Mani S, et al.[37] | support vector machine (SVM), naive Bayes (NB), averaged one dependence estimators (AODE), K-nearest neighbor, decision tree, random forests (RF), logistic regression (LR), lazy Bayesian rules (LBR) | Atyp Lymphs, Ax Temp, Baso (ABS), Base Excess Arterial, Base Excess Venous, Basophils, Base Excess Capillary, Bicarbonate, PCO2 Capillary, pH Capillary, Bilirubin Conjugated, CMB Temperature, C-Reactive Protein, CUM Transfusion, Calcium Ionized, Creatinine Blood, EO Automated Abs, Eosinophil , Glucose Whole Blood, Glucose Blood, etc. | 299 | Single center | Infants | AUROC, sensitivity, specificity, PPV, NPV |
| Shahin J, et al.[38] | multivariable logistic regression models | Age, sex, Severe comorbidities, APACHE lI Score, ICNARC Physiology Score, Primary reason for admission to the critical care unit, Length of stay , Critical care unit stay, etc. | 60, 778 | Multi center | Adults | c index, Brier’s score |
| Sauthier MS, et al.[40] | random forests machine learning algorithms and logistic regression | Age, Gender, ICU days, Pediatric Risk of Mortality II score, Hospital mortality, Highest hypoxemia severity (days 1 and 2), None or minimal, Mild, Moderate, Severe, Arterial blood sample, etc. | 258 | Multi center | Children | AUROC |
| Messinger AI, et al.[39] | Neural networks | charted PAS score, respiratory support and medications | 128 | Single center | Children | median absolute error |
| Le S, et al.[41] | gradient boosted tree models | Age, Antibiotics, Bilirubine, Blood Culture, Creatinine, Diastolic BP, Fluid Bolus, GCS, HR, INR, Lactatey MAP, Organ Dysfunctione PP, Platelets+Resp.Ratev SpO2, Systolic BP, Temp, Urine Outpute WBC, pH | 9, 251 | Single center, MIMIC III | Adults | AUROC, Sensitivity, Specificity, F1, DOR, LR+, LR-, Accuracy, Recall |
| Hsu JC, et al.[42] | SVM embedded with a radial basis function (RBF) kernel. | APACHE II Score, GSC, blood biochemistry test (BUN, Cr, Albumin, Hemoglobin), days using mechanical ventilator, ventilatory variable, arterial PaCo2, and PaO2/FiO2. The respiratory variables, including minute ventilation, repertory rate (f), tidal volume (VT), and P0.1 (pressure of 0.1 second after starting expiration), displayed on the ventilator were recorded at the first minute, 30th minute, 60th minute of the SBT. | 380 | Single center | Adults | Sensitivity, accuracy, specificity, log2C, Log2g |
| Miu T, et al.[43] | logistic regression | Tidal volume, Breathing frequency, Minute ventilation, PEEP, FIO2, SpO2, Suctioning frequency, Secretions quantity score, Positive cuff leak, Heart rate, Systolic blood pressure, Diastolic blood pressure, Mean arterial pressure, Glasgow coma score, Positive cough, Positive gag, Positive corneal, Pupil size, Pupil reaction, Admission SAPS II, Number of prior spontaneous breathing trials, pH, PaCO2, PaO2, PaO2/FIO2, Intracranial pressure, oxygenation | 2007 | Single center | Adults | auroc |
| Isbister GK, et al.[44] | A fully Bayesian approach using logistic regression and time-to-event analysis | patient demographics (age, sex), ingestion details [amount (mg) ingested and estimated time of ingestion], major interventions (mechanical ventilation and duration of ventilation, administration and time of SDAC) and cardiovascularsupport (intravenous fluids, inotropes), etc. | 176 | Single center | Adults | Adjusted odds ratios |
| Ghazal S, et al.[45] | ANN classifier, Bootstrap aggregation of complex decision trees | Age, Weight, Heart Rate, Pulse, Ventilator settings, FiO2, PEEP, Vt, PS above PEEP, PC above PEEP, Ventilator measures, Expiratory minute volume, 1/E ratio, Measured RR, Mean airway pressure, Peak airway pressure | 610 | Single center | Adults | Precision, Recall, F-score |
| Rodríguez A, et al.[46] | CHAID decision-tree analysis | age, sex, comorbidities, Sequential Organ Failure Assessment (SOFA) score, time between symptom onset and ICU or hospital admission, laboratory testing (hemoglobin, count of leukocytes, serum creatinine, etc. | 1, 898 | Multi center | Adults | sensitivity, specificity, positive and negative predictive values, and positive or negative likelihood ratio. |
| Lin PC, et al.[47] | xgboost | Sex, age, weight, Glasgow Coma Scale, temperature (Celsius), heart rate, systolic blood pressure, diastolic blood pressure, respiratory rate, BUN, creatinine, hemoglobin, WBC count, platelet count, INR, total bilirubin, Hb, pH, SpO2, FiO2, PaCO2, HCO3, CO2 (mEqL), lactate, fluid input, fluid balance, urine output | 19275 | Single center, MIMIC-III | Adults | AUC, Sensitivity, Specificity, Precision, F-score, Accuracy |
| Pappada SM, et al.[48] | Artificial Neural Network | 24 Hour time stamp, Real-time Sensor Blood Glucose Value, POC Blood Glucose Value, Hours since last POC Value, Blood Oxygen Saturation, Heart Rate, Respiration Rate, Systolic BP, Diastolic BP, etc. | 127 | Single center | Adults | the percentage of the mean absolute difference (MAD%) |
| Mamandipoor B, et al.[49] | Lasso regression, Random Forest, LSTM | gender, age, ethnicity, admission weight, Respiratory Rate, O2 Saturation, FiO2, glucose, potassium, sodium, Hgb, chloride, creatinine, BUN, bicarbonate, LPM O2, etc. | 13464 | Multi center | Adults | Mean Absolute Error (MAE), Root Mean Squared Error (RMSE), and R-squared (R2) |
| Su L, et al.[50] | random forest, support vector machine, adaptive boosting(AdaBoost), extreme gradient boosting, and shallow neural network | age, ethnicity, gender, initial heparin dose, interval between initial heparin injection and first measurement of activated partial thromboplastin time, creatinine concentration, type of admission, the aspartate aminotransferase to alanine, aminotransferase ratio (AST/ALT ratio) | 3364 | Multi center | Adults | precision, recall, F1 score, accuracy |
| Yu L, et al.[51] | convolutional neural network (CNN) | Na (sodium), K (potassium), Cl (chloride) and HCO3 (serum bicarbonate), Ca (total calcium), Mg (magnesium) and PO4 (phosphate), BUN (blood urea nitrogen), Cr (createnine), Hgb (hemoglobin), Plt (platelet count), WBC (white blood count) | 41113 | Single center, MIMIC III | Adults | Accuracy |
| Cismondi F, et al.[52] | fuzzy modeling | heart rate, oxygen saturation, respiratory rate, temperature, blood pressure, urine collections, infusion products and transfusions.calcium, PTT, hematocrit, fibrinogen, lactate, platelets, INR, hemoglobin. | 746 | Single center, MIMIC II | Adults | accuracy, Sensitivity, specificity |
| Li K, et al.[53] | random forest or logistic regression | age and gender, Admission HR divided by SBP | 1385 | Single center | Adults | The area under the receiver operating characteristic curve (AUROC), classification accuracy, precision, F1 score, and recall |
| Oh SH, et al.[54] | logistic regression | age, medications, altered levels of consciousness, dependent physical activity, intake and output imbalance, abnormal blood pressure and pulse, PaO2, glucose levels, white blood cell counts, platelet counts, levels of potassium, phosphorus, magnesium, low-density lipoprotein, total protein | 1835 | Single center | Adults | degree of conformity, sensitivity, specificity, negative predictive value, auroc, accuracy, Kappa |
| Milbrandt EB, et al.[55] | logistic regression | Subjects, Age, Race, Gender, Comorbidity, Diabetes, Pulmonary disease, Renal disease, Liver disease, Peripheral vascular disease, Cerebrovascular disease, Myocardial infarction, BMI, Medical, Emergency surgery , Trauma case, Admission diagnosis, Cardiovascular, Respiratory, Gastrointestinal, Neurologic, Sepsis, Nonoperative trauma, Metabolic, Renal, Other medical, Surgical, Other surgical, APACHE lI score, ICU LOS, Hospital LOS days, Hospital mortality , Physiologic and therapeutic measures within 6 hours of ICU admission Hemoglobin, Lactate more than 1.5 mg/dl, Creatinine at least 1.6 mg/dl , INR more than 1.9, Inotropes, Mechanical ventilation , Transfusion | 5, 170 | Single center | Adults | receiver operating characteristic curve areas |
| Fialho AS, et al.[56] | Fuzzy Modeling | Arterial base excess, Lactic Acid, Platelets, Sodium, Non-invasive systolic blood pressure, White blood cells, SOFA, BUN, Creatinine SpO2, Temperature | 2, 944 | Single center, MIMIC II | Adults | Values of AUC, sensitivity, specificity and goodness of fit |

### Appendix III: Outcome Evaluation and Prognostic Assessment

| **Article Refs** | **Learning Models** | **Variables** | **Cohort Size** | **Single center/ Multi center** | **Infants/Children/Adults** | **Evaluation Methods** |
| --- | --- | --- | --- | --- | --- | --- |
| Ghose S, et al.[57] | Random Forest | age, gender, height, weight, ICU type, Blood Pressure(diastolic, mean, systolic), Albumin, Alkaline phosphate, Alkaline transaminase, Aspartate transaminase, Bilirubin, Blood urea nitrogen, Cholesterol, Creatinine, Fractional inspired oxygen, Glasgow Coma Score, Glucose, Serum bicarbonate, Hematocrit, Heart rate, Serum potassium, Lactate, Serum magnesium, Mechanical ventilation, Serum sodium, PaCo2, PaO2, pH, Platelets, Respiration rate, SaO2, Temperature, Troponin-I, Troponin-T, Urine output, WBC | 4000 | Single center, a publicly available dataset released by the PhysioNet challenge | Adults | TPR, FPR, PPV, NPV, accuracy, auroc |
| Venugopalan J, et al.[58] | logistic regression,  feedforward neural networks and conditional random fields | Gender, Age, Height, Weight, Ethnicity, Comorbidity, Urea, Albumin, Bilirubin, Creatinine, Sodium, HR, BP, Arterial PH, Arterial PaCO2, Arterial PaO2, etc. | 32, 331 | Single center, MIMIC-II | Adults | Matthews correlation coefficient (MCC) and accuracy |
| Ting HW, et al.[59] | Linear regression models | Sex, age, Alive/dead, Admission type, Disease type | 154 | Single center | Adults | t test, receiver operating characteristic (ROC) curve and Wilcoxon signed rank test |
| Sha Y, et al.[60] | logistic regression, SVM, RNN | sequential diagnostic codes | 7, 537 | Single center, MIMIC III | Adults | Matthews correlation coefficient, auroc, F1 score |
| Meiring C, et al. [61] | logistic regression, random forest, adaboost, support vector machine, neural network | Age, sex, admission, APACHE-II score, adrenaline (epinephrine), noradrenaline (norepinephrine) , vasopressin, mechanical ventilation, heart rate (HR), arterial pressure (MAP), PaO2/FiO2 ratio, sodium, potassium,  lactate, creatinine, CRP, pH | 22, 514 | Single center | Adults | AUC |
| Bukan RI, et al. [62] | logistic regression model | age, sex, excessive alcohol consumption, medical or surgical background, APACHE II score, 2 SF-36 and SF-12 scores | 318 patients | Single center | Adults | AUC |
| Hsieh YZ, et al.[63] | Neural Network, SVM | age, systolic arterial blood pressures (SABP), diastolic ABP (D-ABP), mean ABP (M-ABP), systolic noninvasive blood pressures (S-NBP), diastolic NBP (D-NBP), mean NBP (M-NBP), respiratory rate (RR), heart rate (HR), body temperature (BT) | 300 | Single center | Adults | accuracy, Confidence, Sensitivity, ROC curve |
| Oeyen S, et al.[64] | multivariate linear regression models, LASSO | demographics, hospital days prior to ICU admission, living and work circumstances before ICU admission, hospitalization in the last 6 months, comorbidity, main ICU admission diagnosis, etc. | 1953 | Single center | Adults | R^2^ ,adjusted R^2^ and the root of the cross-validated prediction error |
| de Lange DW, et al.[65] | Multivariable logistic regression | age, sex, reason for ICU admission categorized into 11 options, vasoactive drugs,  Clinical Frailty Scale, SOFA score, intubation with mechanical ventilation, RRT | 3730 | Multi center | Adults | AUC, Brier score |
| Guidet B, et al.[66] | Principal Component Analysis, multivariable model | age, SOFA score, CPS, IQ code, Katz’s scale and clinical frailty scale | 3920 | Multi center | Adults | HR (95%CI), p |
| Heyland DK, et al.[67] | multivariable logistic regression | Baseline PPS, Baseline Short Form-36 Physical Function, Age, Sex, Maried, Acute Physiology and Chronic Health Evaluation lI, Baseline Sequential Organ Failure Assessment score, Medical admission, Charlson Comorbidity Index, Functional Comorbidity Index, Primary diagnosis of trauma, Primary diagnosis of sepsis, Primary diagnosis of stroke, Primary diagnosis of emergency coronary artery bypass grafting or valve replacement, Dementia, Clinical frailty scale, Baseline Informant Questionnaire on Cognitive Decline in the Elderly ≥3.6 | 434 | Multi center | Adults | Odds Ratio, c, p |
| Puskarich M, et al.[68] | decision tree | age, chronic disease, lactate and five biomarkers | Not available | Multi center | Adults | sensitivity, specificity, positive predictive value, negative predictive value, auroc |
| Wong HR, et al.[69] | decision tree | five candidate biomarkers, admission lactate concentration, age, and chronic disease burden | 881 | Multi center | Adults | auroc, sensitivity, specificity, positive predictive value, negative predictive value |
| Jaimes F, et al.[70] | logistic regression and neural networks | age, immunosuppressive systemic disease, general systemic disease, Shock Index, temperature, respiratory rate, Glasgow Coma Scale score, leucocyte counts, platelet counts, creatinine | 533 patients | Multi center | Adults | ROC curve and the Hosmer-Lemeshow goodness-of-fit test |
| Ribas Ripoll VJ, et al.[71] | soft-margin support vector machines Quotient Basis Kernel (QBK) | Gender, Age, ICU length of stay, Mechanical ventilation, Invasive blood pressure, Vasoactive medications, ICU mortality, SOFA , SAPS I | 400 | Single center, MIMIC II | Adults | correct rate, sensitivity, specificity, AUC |
| Sha Y, et al.[72] | Smith Waterman | hematocrit, potassium, sodium, creatinine, platelets, urea nitrogen, chloride, bicarbonate, anion gap, leukocytes, point-of-care (POC) glucose, oxygen saturation, arterial POC pH, arterial POC pCO2, arterial POC pO2, sodium, POC ionized calcium, potassium, calcium, glucose. | 22, 870 from MIMIC-II;  4, 975 from CHOA | Multi center | Children | sensitivity and F-measure |
| Yang T, et al.[73] | logistic regression | diabetes, APACHE II score, serum osteopontin, serum interleukin 6, serum interleukin 18, urine output, urinary creatinine, serum neutrophil gelatinase-associated lipocalin, urinary IL-18, serum cystatin C, serum creatinine, urinary neutrophil gelatinase-associated lipocalin | 102 | Single center | Adults | auroc, sensitivity, specificity |
| Xu Z,  et al.[74] | Logistic Regression (LR), L2 norm regularized Logistic Regression  (Ridge), Random Forest (RF), and Gradient Boosting  Decision Tree (GBDT) | Demographics, Medications, Comorbidities, Chart-events, Lab-events | 58,976 | Single center, MIMIC III | Adults | AUC, recall and precision |
| Trongtrakul K, et al.[75] | logistic regression | age, SOFA non-renal score, sepsis, emergency surgery, perioperative blood loss, perioperative urine output | 3474 | Single center | Adults | auroc, C-statistic, sensitivity, specificity, positive predictive value (PPV), and negative predictive value(NPV) |
| Bernal W, et al.[76] | Cox proportional hazards model | Age, Sex, Hepatic encephalopathy grade, Glasgow coma scale score, Cardiovascular failure, Mean arterial pressure, INR, Bilirubin, AST, Creatinine, Arterial pH, Arterial lactate | derivation set (n=350) and an validation set (n=150) and external validation dataset (n=412) | Multi center | Adults | area under receiver operating characteristic curve (AUROC), root mean square error (RMSE) |
| Lindenmeyer CC, et al.[77] | logistic regression analysis | Age, gender, co-morbidities, etiology of chronic liver disease, vital sign，platelet count, prothrombin time (PT), International normalized ratio, lactate, arterial blood gas, pH, 24-hour urine output, need for mechanical ventilation, etc. | 436 | Single center | Adults | AUROC |
| Balekian AA, et al. [78] | multivariable logistic regression | Male, Age in years, Race, APACHEII, MELD, MV, Dialysis, Sepsis, GI Bleed, Alcohol use, Direct ICU Admit, ICU LOS in days, Home discharge, Hospital transfe, SNF discharge, Left AMA, Hospice, Expired | 653 | Multi center | Adults | AUROC |
| Santos HGD, et al.[79] | logistic regression, penalized logistic regression, artificial neural networks, basic decision trees, random forests, gradient boosted trees | Age, sex, BMI, Chronic renal failure, Chronic pulmonary failure, Chronic heart failure, Diabetes, Alcoholism, Use of steroids, Smoking, Estimated Glasgow Coma score, Total bilirubin, Body temperature, Creatinine, Heart rate, Leukocytes, pH, Platelets, Noradrenaline use, Average pressure, ECOG performance status, Delirium, Type of admission, Nosocomial infection, Respiratory infection, Invasive mechanical ventilation, health history related to cancer disease, current cancerrelated complications | 777 | Multi center | Adults | auroc, confusion matrix |
| Vincent F, et al.[80] | Logistic regression analyses | Systemic extension of the disease, Underlying tumor, Renal replacement therapy during ICU, Vasopressors, Mechanical ventilation | 1, 053 | Multi center | Adults | sensitivity, specificity, ROC curve |
| Lee S, et al.[81] | random forest classifiers | Age, Sex, Race, Hispanic, In-hospital cardiac arrest location, Witnessed cardiac arrest, Bystander CPR for out-of-hospital cardiac arrest, CPR duration, Initial rhythm, Asystole, Pulseless electrical activity, Bradycardia, Ventricular fibrillation or tachycardia, other/unknown, Cardiac arrest cause, Sudden infant death syndrome, Drowning, Shock, Respiratory failure, Trauma, Initial lactate, Lowest pH initial 24 h after cardiac arrest, Intubated, Induced hypothermia, Benzodiazepine infusion, Length of stay: ICU, Length of stay: hospital, Early EEG, Late EEG | 87 | Single center | Children | ROC/AUC, Sn/Sp, PPV/NPV |
| Murtuza B, et al.[82] | Conditional inference tree modelling | Weight, Ascending aorta size, Morphologic type, TR>mild, Impaired RV function pre-stage I, Restrictive IAS, Antenatal diagnosis, blood lactate clearance | 221 | Single center | Infants | P value |
| Gracia Arnillas MP, et al.[83] | the recursive partitioning and regression tree (RPART) method | age, sex, Hypertension, Diabetes mellitus, Headache, GCS, Hydrocephalia, Brain edema, Vasospasm, Fisher, Hunt-Hess, APACHE, Endovascular coiling, Neurosurgical clipping, Non treatment. MR-proADM | 90 | Single center | Adults | sensitivity, specificity, ROC curve and AUC |
| Haveman ME, et al.[84] | Random Forest  classifier | Gender, Age in years, Injury Severity Score, ICU stay in days since trauma, EEG start in hours after trauma, EEG recording time in hours, ICP, Decompressive craniectomy, Medication administration, Propofol, Midazolam, Fentanyl, Noradrenaline | 57 | Single center | Adults | AUC |
| Wildman MJ, et al.[85] | multivariate logistic regression | FEV1, Respiratory rate, Weight loss in last 6 months, Katz Activity of Daily Living (ADL)score, BMI, Charlson co-morbidity score, Long term domiciliary oxygen treatment, Previous endotracheal intubation, Admitted to hospital in last 6 months, Ankle oedema, Abnormal shadow on chest X-ray, Reported prior quality of life, Congestive cardiac failure | 832 | Multi center | Adults | Hosmer-Lemeshow chi-sq, Slope CI, c (area under ROC curve), P (different to clinicians) |
| Daly K, et al.[86] | logistic regression model | acute physiology points, length of stay in intensive care, therapeutic intervention score, duration (days) on mechanical ventilation, dialysis, age, presence of chronic ill health, number of failing organs, whether or not the patient had had cardiothoracic surgery | 13924 | Single center | Adults | AUROC, sensitivity, specificity |
| Hernández-Tejedor A, et al.[87] | multi-variate logistic regression analysis | sex, Age, Dependency for basic activities of daily living, APACHE lI at ICU admission, SOFA at ICU admission, SOFA the day before the complication(day -1), SOFA the day of the complication (day 0), ICU mortality, Hospital mortality, ICU length of stay , Hospital length of stay, etc. | 13, 456 | Multi center | Adults | Receiver operating characteristic (ROC) curve, sensitivity, specificity, positive predictive values, negative predictive values |
| Ji SY, et al.[88] | Logistic, AdaBoost, C4.5, CART, SVM, RBF NN | age, gender, blood pressure, cheifcomp, airway, prefluids, GCS, heart rate, respiration rate, ISSHead&Neck, ISS, EDEYE, ED Verbal, EDRT, Head AIS, Thorax AIS, Abdomen AIS, Intubation, Prexcomor, Complications, Safety, Pluse, Position, ChiefComp, Blunt | 2086 | Multi center | Adults | accuracy, auroc |
| Che Z, et al.[89] | Linear Support Vector Machine (SVM), Logistic Regression (LR), Decision Trees (DT), Gradient Boosting Trees (GBT), DNN, GRU, GRU+DNN interpretable mimic learning method | It contains a set of 27 static features such as demographic information and admission diagnoses, and another set of 21 temporal features (recorded daily) PaO2, MAP, BE, FiO2, PF, δPF, PH, PRISM12, PIM2S, VE, VI, etc. | 398 | Single center | Children | auroc, auprc |
| Ebadollahi S,et al.[90] | Similar Patients Metric, Locally Supervised Metric Learning (LSML) | mean ABP measure, systolic ABP, diastolic ABP, Sp02 and heart rate | 1500 | Single center, MIMIC II | Adults | Classification and Retrieval Accuracy, Patient Prognosis Accuracy |
| Castiñeira D, et al.[91] | logistic regressions, random forests, support vector machine approaches, and gradient boosted trees | heart rate, breathing frequency, pulse, SpO2, sex, age, pre-ICU admission location, elective admission, recovery after the procedure, cardiac bypass, diagnosis risk, lack of pupillary response, mechanical ventilation, first systolic blood pressure, base excess, FIO2, PaO2 | 284 | Single center | Children | accuracy, auroc |
| Mueller M, et al.[92] | artificial neural network (ANN) and a multivariate logistic regression model (MLR). | AB(arterial blood gas), AB(cap. blood gas), Balanced pattern(no), Balanced pattern(yes), Extubation failureExtubation success Mode(AC), Mode(SIMV), Overventilated(no), Overventilated(yes), Ethnicity(black), Ethnicity (Other), Ethnicity(white), Sex(female), Sex(male), Age_D, APGAR1, APGAR5_1, BE, BP, CurrWeight, dBE, dBP, dFIO2, dHCO3, dIErat, dINSP, dMAP, dPaCO2dPaO, dPEEP, dPH, dPIP, dPulse, dRATE, dRRatio, dSaO2, dTIME, dVr, FiO2, Gst_age, HCO3, IEratio, INSP, Lag, MAP, Paco2, Pao2, PEEP, pH, PIP, Pulse, Rratio, Rate, Saline, SaO2, Theoph, Vr, TXBETAME | 183 | Multi center | Infants | AUC, ROC |
| Mueller M, et al. [93] | artificial neural network | AB(arterial blood gas), AB(cap. blood gas), Balanced pattern(no), Balanced pattern(yes), Extubation failureExtubation success Mode(AC), Mode(SIMV), Overventilated(no), Overventilated(yes), Ethnicity(black), Ethnicity (Other), Ethnicity(white), Sex(female), Sex(male), Age_D, APGAR1, APGAR5_1, BE, BP, CurrWeight, dBE, dBP, dFIO2, dHCO3, dIErat, dINSP, dMAP, dPaCO2dPaO, dPEEP, dPH, dPIP, dPulse, dRATE, dRRatio, dSaO2, dTIME, dVr, FiO2, Gst_age, HCO3, IEratio, INSP, Lag, MAP, Paco2, Pao2, PEEP, pH, PIP, Pulse, Rratio, Rate, Saline, SaO2, Theoph, Vr, TXBETAME | 183 | Multi center | Infants | area under the ROC curve |
| Dunning J, et al.[94] | logistic regression, Recursive partitioning | Parsonnet score, LVEF, age, PA systolic pressure, Emergency re-operation, Current diuretic use for LVF, ejection fraction, IV nitrates, Parsonnet increment, creatinine, Redo operation | 6991 | Multi center | Adults | auroc, Sensitivity , Specificity, negative predictive value |
| Manji RA et al.[95] | Logistic regression | Age, Sex, Peripheral vascular disease, Renal insufficiency(creatinine >1.8mg/dL), Renal failure(dialysis), Cerebrovascular disease, Preoperative infection, Operative variables, Procedure, Isolated CABG, Single non-CABG procedure, Nonelective procedure, Selected ICU variables, Postoperative cardiac arrest, ECMO/VAD after cardiotomy, Cerebrovascular accident, Adult respiratory distress syndrome, Number of ventilation d(within first 10 d), Septic shock, Central line infection, Upper gastrointestinal bleeding, Acute renal failure(dialysis needed), ICULOS(d), Categorical variables, Preoperative renal failure(dialysis), ECMO/VAD after cardiotomy, Preoperative infection, Preoperative renal insufficiency (creatinine>1.8mg/dL), Cerebrovascular disease, Peripheral vascular disease, Female sex, Continuous variables, ICULOS beyond 5 d, Mechanical ventilation, Cardiopulmonary bypass time(per 30 min increase in bypass time), Ejection fraction grade(increase of 1 grader | 9, 545 admissions | Multi center | Adults | Odds Ratio, 95% Confidence Interva, l p Value |
| Brandi S, et al.[96] | equations of generalized estimation with binomial distribution, logistic function linking and structure of autoregressive correlation, multiple logistic model | age, sex, indication for admission (elective, urgency, emergency), type of admission, outcome (transference, hospital discharge, discharge with homecare, or death), the use of mechanical ventilation, origin (pediatric unit, emergency unit, surgical center, pediatric outpatientunit, bone marrow transplantation or external), readmission within 48 hours, reason for admission (respiratory failure, sepsis, shock, post-operatory, liver failure, neurology, hemodynamic monitoring, post-event monitoring, other), Paediatric Logistic Organ Dysfunction (PELOD) score, presence of venous access | 1, 815 consecutive admissions | Single center | Children | AUROC |
| McWilliams CJ, et al.[97] | random forest, Logistic regression | Gender, Age, BMI, Length of stay, Discharge delay, In-hospital mortality, Readmission to ICU, Negative outcomes, airway, fio2, spo2, hco3, resp, bp, hr, pain, gcs, temp, haemoglobin, k, na, creatinine, bun | 7592+1870 | Multi center | Adults | AUROC, Accuracy, F1, Specificity, pAUROC, Brier, Sensitivity |
| Lin YW, et al.[98] | LSTM | Glasgow coma scale eye opening, Glasgow coma scale verbal response, Glasgow coma scale motor response, Glasgow coma scale total, Capillary refill rate, Diastolic blood pressure, Systolic blood pressure, Mean blood pressure, Heart Rate, Glucose, Fraction inspired oxygen, Oxygen saturation, Respiratory rate, Body Temperature, pH, Weight, Height, Gender, Age, Insurance Type, Race, Chronic diseases | 35334 | Single center, MIMIC-III | Adults | Accuracy, Precision, Recall, Sensitivity, AUROC, AUPRC |
| Czeiter E,  et al.[99] | Univariable and multivariable analysis | S100B, neuron-specific enolase (NSE), GFAP, UCH-L1, neurofilament protein-light (NFL), and total tau (t-tau) | 2867 | Multi center | Adults | AUROC |
| Yin W, et al.[100] | bivariate logistic regression model | Gender, Age, APACHE II, Heart rate, Systolic blood pressure, Diastolic blood pressure, Mean blood pressure, Urine output per hour, Respiratory rate, PaO2/FiO2, Ventilation/Non-ventilation, PEEP, Length of mechanical ventilation, ICU length of stay, Hospital length of stay, ICU mortality, Hospital mortality, Respiratory disease, Severe pneumonia, AECOPD, ARDS, Others, Shock, Cardiac arrest, Heart failure, Renal failure, Liver failure, Acute obstructive suppurative cholangitis, Severe acute pancreatitis, Acute peritonitis, Bowel obstruction, Multiple trauma, Tumor, Stroke, CNS infection, Postoperative patients, Organ Transplantation, Burn | 451 | Single center | Adults |  |
| Shickel B, et al.[101] | recurrent neural network (RNN) | MAP, Dopamine, Dobutamine, Epinephrine, Norepinephine, FiO2, PaO2, SpO2, Ventilation, Creatinine, Urine, GCS, Bilirubin, Platelets | 27660/35, 993 | Multi center | Adults | AUC |
| McRae MP, et al.[102] | lasso logistic regression model | Age, Sex, cTnI, CK-MB, MYO, CRP, PCT | 160 | Multi center | Adults | AUC |

### Appendix IV: Treatment Decisions

| **Article Refs** | **Learning Models** | **Variables** | **Cohort Size** | **Single center/ Multi center** | **Infants/Children/Adults** | **Evaluation Methods** |
| --- | --- | --- | --- | --- | --- | --- |
| Srinivasan S, et al.[3] | Reinforcement Learning | age, weight, SOFA, OASIS and SAPS scores, gender, ethnicity, emergency, admission urgency and hours from admit to the ICU. bicarbonate, bun, creatinine, fio2, glucose, hct, heart rate, lactate, magnesium, meanbp, platelets, potassium, sodium, spo2, spontaneousrr, temp, urine, wbc. normalized vasopressor dosages and fluid boluses. | 16502 | Single center, MIMIC III | Adults | action matching, confusion matrices |
| Yu C, et al.[4] | Reinforcement Learning | respiration rate, heart rate, arterial pH, positive end-expiratory pressure (PEEP) set, oxygen saturation pulse oxymetry (SpO2), inspired oxygen fraction (FiO2), arterial oxygen partial pressure, plateau pressure, average airway pressure, mean non-invasive blood pressure, body weight (kg), age, and ventilation. | 707 | Single center, MIMIC III | Adults | convergence, learning efficiency, consistency with the true policies of the doctors |
| Nemati S, et al.[5] | Reinforcement Learning | aPTT, arterial carbon dioxide level (CO2), heart rate (HR), heparin dose, albumin, SBP and DBP, bilirubin, creatinine, Glasgow Coma Score (GCS), hematocrit, hemoglobin, International normalized ratio of prothrombin (INR), blood PH, platelet count, prothrombin time, respiration rate, oxygen saturation of arterial blood (SA02), SOFA scores, temperature, troponin, urea, white blood cell count (WBC), ethnicity, ICU service type, gender, transfer from another hospital, pulmonary embolism, obesity, age, weight. | 4470 | Single center, MIMIC II | Adults | accumulated reward, time taken to enter the therapeutic range, long-term performance |
| Yu C, et al.[6] | Reinforcement Learning | respiration rate, heart rate, arterial pH, positive end-expiratory pressure (PEEP) set, oxygen saturation pulse oxymetry (SpO2), inspired oxygen fraction (FiO2), arterial oxygen partial pressure, plateau pressure, average airway pressure, mean non-invasive blood pressure, body weight (kg), age, and ventilation. | 8860 | Single center, MIMIC III | Adults | Accuracy rate, mean square error |
| Komorowski M, et al.[7] | Reinforcement Learning | Age, Gender, Weight, Readmission to intensive care, Elixhauser score, Modified SOFA*, SIRS, Glasgow coma scale, Heart rate, systolic, mean and diastolic, blood pressure, shock index, Respiratory rate, SpO2, Temperature, Potassium, sodium, chloride, Glucose, BUN, creatinine, Magnesium, calcium, ionized calcium, carbon dioxide, SGOT, SGPT, total bilirubin, albumin, Hemoglobin, White blood cells count, platelets count, PTT, PT, INR, pH, PaO2, PaCO2, base excess, bicarbonate, lactate, PaO2/FiO2 ratio, Mechanical ventilation, FiO2, Current IV fluid intake over 4h, Maximum dose of vasopressor over 4h, Urine output over 4h, Cumulated fluid balance since admission, Hospital mortality, 90-day mortality | 96156 | Multi center | Adults | Good model calibration was confirmed by plotting the relationship between the return of the clinicians’ policy and patients’ 90-day mortality. |
| Lopez-Martinez D, et al. [8] | Reinforcement Learning | Pain scores, Morphine interventions, Non-morphine analgesic interventions, Physiological signals | 38600 | Single center, MIMIC III | Adults | physician actions against model actions |
